# Windswept hip deformity in children with cerebral palsy: a population-based prospective follow-up

**DOI:** 10.1007/s11832-016-0749-1

**Published:** 2016-06-18

**Authors:** Gunnar Hägglund, Henrik Lauge-Pedersen, Måns Persson Bunke, Elisabet Rodby-Bousquet

**Affiliations:** Department of Clinical Sciences, Lund, Orthopaedics, Lund University, 22185 Lund, Sweden; Centre for Clinical Research, Uppsala University, County Hospital, 72189 Västerås, Sweden

**Keywords:** Cerebral palsy, Windswept hip deformity, Hip dislocation, Scoliosis

## Abstract

**Purpose:**

To analyze the development of windswept hip deformity (WS) in a total population of children with cerebral palsy (CP) up to 20 years of age, the association between WS and hip dislocation, and femoral varus osteotomy and scoliosis, and the impact of a hip surveillance program on the subsequent incidence of WS.

**Methods:**

This is a prospective study on children with CP in southern Sweden included in the Swedish follow-up programme and registry for CP (CPUP). All children born between 1990 and 1995 with CP were included; those born between 1990 and 1991 did not partake in the hip surveillance program until they were older (3–5 years of age) and served as a historic control group. Children born between 1992 and 1995 were included in the hip surveillance program from about 2 years of age and constituted the study group.

**Results:**

In the control group, 12 of 68 children (18 %) developed WS. In the study group of 139 children, 13 (9 %) developed WS (p = 0.071). Of all 25 children with WS, 21 also developed scoliosis and 5 developed a hip dislocation. The number of children with WS starting in the lower extremities was significantly lower in the study group (*p* = 0.028). No difference between the two groups was seen regarding WS that started in combination with scoliosis.

**Conclusion:**

With early inclusion in a hip surveillance program and early treatment of contractures, it appears possible to reduce the frequency of WS starting in the lower extremities.

## Introduction

Children with cerebral palsy (CP) exhibit spasticity, muscle weakness, and immobility, in combination with an inability to deal with the effects of gravity. Thus, these children are at risk of developing muscle contractures, hip dislocation, windswept hip deformity (WS), and scoliosis, either in isolation or in combination. The WS pathology comprises abduction and external rotation of one hip, with the opposite hip in adduction and internal rotation. WS is a severe problem that is difficult to treat, and it impairs the child’s standing ability and interferes with comfort when lying and sitting [1].

A CP registry and healthcare program (CPUP) aimed at preventing hip dislocation and severe contractures was initiated in southern Sweden in 1994 [2, 3], and, currently, more than 95 % of the total population of children with CP nationwide has been included in the program [4]. The original cohort from southern Sweden consisting of the total population of children with a verified diagnosis of CP in this area has now been followed prospectively for more than 20 years. In 2005, we analyzed the prevalence of WS in this cohort, as well as the outcomes of the hip surveillance program [5]. The frequency of WS was 12 % in the historical control group, which included the children who had not participated in the hip surveillance program until they were 3–5 years of age, and 7 % in the study group, who had participated in the hip prevention program from an earlier age. No child with WS in the study group had a hip dislocation, compared with 9 % in the control group.

In the present study, we reevaluated the same cohort as young adults and the aims were:

1. To analyze any further development of WS between 10 and 20 years of age;
2. To analyze associations between WS and hip dislocation, femoral varus osteotomy, and/or scoliosis; and
3. To analyze the impact of the CPUP hip surveillance program on the subsequent incidence of WS.

## Subjects and methods

We performed a prospective study on children with CP who were born and residing in southern Sweden and included in the Swedish CPUP registry. All children born in 1990–1995 with CP were included; those born in 1990–1991 did not partake in the hip surveillance program until they were older (3–5 years of age) and served as a historic control group in this study. Children born in 1992 or later, however, were included in the hip surveillance program at an earlier age than the controls. Only those born in the catchment area (or who moved into the area before 2 years of age) and participated in the CPUP were included. The diagnosis of CP was verified by a neuropediatrician after each child’s fourth birthday. All subtypes of CP were included and classified according to the Surveillance of Cerebral Palsy in Europe (SCPE) criteria [6]. Gross motor function was classified by the child’s physiotherapist according to the Gross Motor Function Classification System (GMFCS) [7]. Although some children had died or moved out of the area during the follow-up period, available data from all children were included in the current analysis.

The CPUP includes a standardized individual follow-up of gross motor function, clinical findings, and treatment. Each child’s physiotherapist performs a clinical examination and fills out an assessment form twice a year until the child turns 6 years old, and then once a year. The clinical examination includes measurements of the range of joint motion using a goniometer according to a manual (http://www.cpup.se) and a clinical examination of the spine. The goal of the standardized follow-up is early detection of contractures and deformities that will then allow for early, preferably non-surgical, treatments. These treatments consist of postural management, provision of orthotics and assistive devices, individualized training, and treatment to reduce spasticity when needed. Most children in GMFCS levels III–V receive a customized standing brace. All children born in 1990 or later, i.e., both the control and the study groups, were included in standardized follow-ups performed by physiotherapists from 1994 onwards. Thus, children in the control group were enrolled in the CPUP at 3–5 years of age, compared to those in the study group, who participated in the program starting at 2 years of age.

Children in the CPUP born 1992 or later also undergo a radiographic follow-up of the hips. Those at GMFCS levels III–V have annual examinations until 8 years of age. After that, radiographs are performed on an individual as-needed basis. The migration percentage (MP) [8] is measured on all radiographs. Hips at risk for dislocation, usually hips with MP >40 %, are treated with adductor–psoas tenotomy, varus osteotomy of the proximal femur, or pelvic reconstruction, usually with Dega osteotomy [9]. In this study, a hip dislocation was defined as an MP of 100 %. Children in the control group were examined radiographically when they were included in the clinical follow-up at 3–5 years of age, and after that on an individual as-needed basis. The numbers of children treated with varus osteotomy of the proximal femur were recorded.

Furthermore, all children with moderate or severe scoliosis on clinical examinations were examined radiographically. The examination was performed with the child in a sitting position if possible, otherwise in a supine position. Children with bilateral CP and at least 50 % difference in abduction, internal, and/or external rotation between the left and right hips were defined as having WS, in accordance with the modified definition of that developed by Young et al. [10]. At least two consecutive measurements that met these criteria, with intervals of at least 6 months, were required for the child to be classified as having WS. Children with a Cobb angle of 20° or more were classified as having scoliosis.

### Statistics

Fisher’s exact test was used to compare the total number of children with WS and the number of children with WS who also developed hip dislocation, varus osteotomy, and/or scoliosis at the follow-up at 10 and 20 years of age, respectively.

### Ethics

The Medical Research Ethics Committee of Lund University approved the study (LU-443-99).

## Results

The initial cohort consisted of 214 children who fulfilled the inclusion criteria: 71 were born in 1990–1991 (historical control group) and 143 were born in 1992–1995 (study group). Three children died and four moved before turning 10 years old, leaving 207 children, and an additional six children died and 14 moved prior to the 20-year follow-up, resulting in 187 participants still included and providing data when they were 20 years of age (Table 1). The GMFCS levels and gender distributions of the initial cohort are shown in Table 2. Of the 207 children who were alive and residing in the area at 10 years of age, 12 of the 68 children in the control group (18 %) later developed WS, compared to 13 of the 139 children in the study group (9 %) (Table 2). Two of the children with WS in the control group and three in the study group had died before 20 years of age (Table 3).

**Table 1:** Number of children with cerebral palsy (CP) in the control group (born in 1990–1991) and in the study group (born in 1992–1995)

	Born in 1990–1991	Born in 1992–1995	Total
Born or moved into the area before 2 years of age	71	143	214
Moved out before 10 years of age	1	3	4
Died before 10 years of age	2	1	3
Number of children participating in 10-year FU	68	139	207
Moved out between 10–20 years of age	2	4	6
Died between 10–20 years of age	4	10	14
Number of children participating in 20-year FU	62	125	187

*FU* follow-up

**Table 2:** Distribution of children in terms of gender and Gross Motor Function Classification System (GMFCS) level in the control group (born in 1990–1991) and the study group (born in 1992–1995), shown as *number* (%)

	Born in 1990–1991	Born in 1992–1995	Total
Male	34 (48)	93 (73)	127 (59)
Female	37 (52)	50 (27)	87 (41)
GMFCS level			
I	30 (42)	67 (47)	97 (45)
II	16 (23)	20 (14)	36 (17)
III	11 (15)	16 (11)	27 (13)
IV	7 (10)	21 (15)	28 (13)
V	7 (10)	19 (13)	26 (12)
Total	71 (100)	143 (100)	214 (100)

**Table 3:** Total numbers of children with windswept hip deformity (WS) in the control and study groups at 10 and 20 years of age, and the numbers of these cases with WS combined with scoliosis (S), hip dislocation (HD), or femoral osteotomy (FO)

	Control group		Study group		*p* value	
	10 years (*n* = 68)	20 years (*n* = 62)	10 years (*n* = 139)	20 years (*n* = 125)	10 years	20 years
WS (total)	8	10 + 2*	10	10 + 3*	0.200	0.071
WS + S	6	8 + 2*	4	9 + 2*	0.067	0.100
WS + HD	5	3 + 2*	0	0	0.003	0.003
WS + FO	0	1	8	7 + 2*	0.040	0.100
WS + S + HD	4	3 + 2*	0	0	0.011	0.004
WS + S + FO	0	1	4	6 + 2*	0.200	0.140

* Children with WS (+S, HD, or FO) who died or moved out of the area between the 10- and 20-year follow-up

Of the 25 children with WS (20 boys), 19 had at least a 50 % difference in both abduction and rotation between the right and left hips, two had a 50 % difference in abduction only, and four in rotation only.

WS in combination with scoliosis with a Cobb angle ≥20° was seen in 8 of the 62 children (13 %) in the control group and in 9 of the 125 children (7 %) in the study group (Table 3). Further, two children in the control group and two children in the study group developed WS and scoliosis but died before 20 years of age (Table 3). Of the 21 children with both scoliosis and WS, the curve was convex to the left in 14 children and to the right in seven. One of the children with a left-sided curve in the lumbar spine also had a right-sided thoracic curve. All children with scoliosis had a pelvic obliquity, with the side opposite to the convex lumbar side being higher. In 19 children, the WS hips were directed towards the convex lumbar side, whereas the hips were deviated to the opposite side of the convexity of scoliosis in two children. One of these two children had bilateral hip dislocation. The other child was treated with bilateral varus osteotomy of the proximal femur. The left hip was treated with a second osteotomy 3 years later. Postoperatively, the child had both hips deviated to the right side, opposite to the convexity of scoliosis. Scoliosis with a Cobb angle ≥20° was seen in a further four children without WS in the control group and in ten children in the study group.

WS in combination with hip dislocation was seen in five children in the control group, of whom two died before 20 years of age, but none in the study group (*p* = 0.003). Two children in the control group and one child in the study group developed hip dislocation without WS. The child in the study group was included in the hip prevention program, but was considered to be in too poor general condition to undergo preventive surgery.

Varisation osteotomy of the proximal femur was performed in four children in the control group and in 18 in the study group. In the control group, one child had WS diagnosed before the osteotomy and the other three did not have WS. In the study group, three children had no WS preoperatively and developed WS directly following surgery. Of the remaining 15 children, six had WS diagnosed before the osteotomy and nine had no WS (Table 3).

Of the 25 children with WS, nine developed WS after—or simultaneously with—scoliosis. In 13 children, WS was seen in isolation or after the hip dislocated, and WS developed after femoral varus osteotomy in three children. Excluding those hips in which WS appeared because of varus osteotomy, the number of children with WS starting in the lower extremities (Table 4) was significantly reduced in the study group (*p* = 0.028). No difference between the two groups was seen regarding the incidence of WS in combination with scoliosis. At the time of WS diagnosis, the children in the control group had a mean knee flexion contracture of −19° (range −35° to −10°) and those in the study group had a mean contracture of −12° (range −30° to 0°).

**Table 4:** Numbers of children with WS associated with the deformity first identified

Deformity first identified	Control group, *n* (%)	Study group, *n* (%)
Windswept hip deformity	4 (6)	5 (3.5)
Hip dislocation	4 (6)	0 (0)
Femoral osteotomy	0 (0)	3 (2)
Scoliosis	4 (6)	5 (3.5)

Proportions of children are based on the numbers of children in the control (*n* = 71) and study (*n* = 143) groups, respectively

## Discussion

**Fig. 1:**
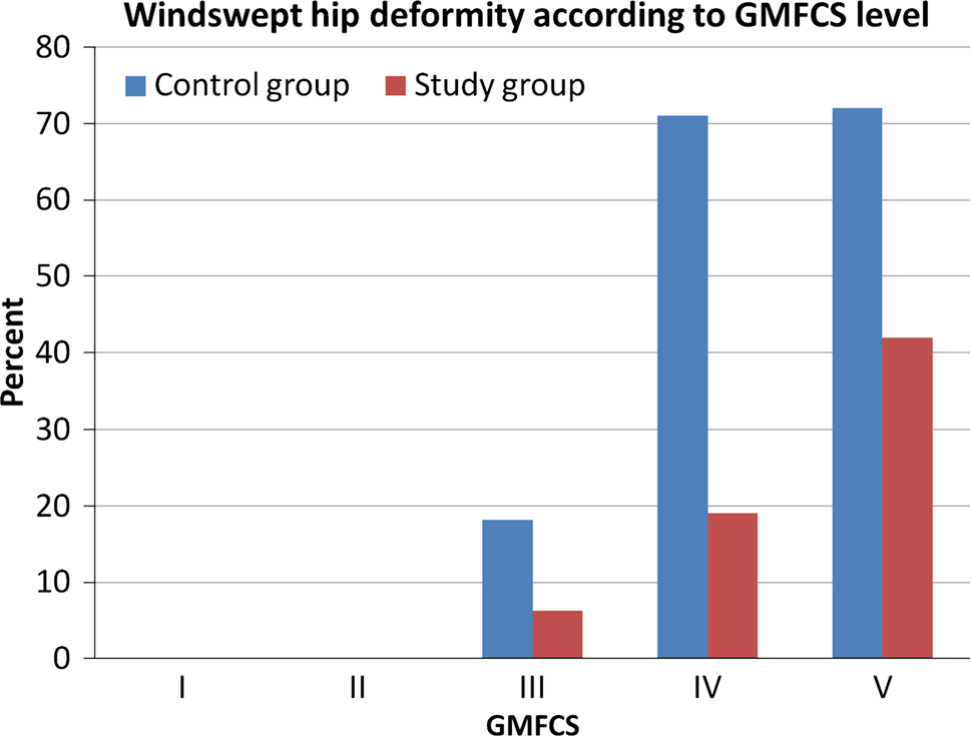
Percentages of children with windswept hip deformity (WS) in the total population of children with cerebral palsy (CP) born in 1990–1991 (control group, *n* = 71) and in 1992–1995 (study group, *n* = 143), according to the levels of Gross Motor Function Classification System (GMFCS)

The frequency of WS increased from 12 % in the control group at 10 years of age to 18 % at 20 years of age and from 7 to 9 % in the study group. The frequency of WS was associated with gross motor function, with 52 % of those at GMFCS level V being affected (Fig. 1). The proportion of children with WS starting in the lower extremity was lower in the study group, whereas the numbers of children with WS starting from the spine were the same in both groups. The only known difference between the groups was that those in the study group were included in the surveillance program early, often before the age of 2 years, which highlights the importance of initiating the standardized follow-up of children with CP very early.

There were several limitations to this study. The number of children with WS was relatively small, which could be why few of the comparisons were statistically significant. However, the strength of the study is that it covered a total population followed prospectively in a standardized manner. Some of the children who died or moved out of the area during the follow-up period might have developed WS at a later stage. Nevertheless, the proportions of children who died or moved were similar between the two groups. The referral for radiographic examinations of scoliosis were based on the physiotherapists’ clinical examination of the spine. Some children with a Cobb angle >20° might have been classified as having mild scoliosis and were not examined radiographically. However, a validation study of the clinical examinations performed in the CPUP has shown a high sensitivity and specificity and a high inter-rater reliability to select children with a Cobb angle ≥20° [11].

The risk of hip dislocation is highest in young children with CP [12]; therefore, most preventive surgery is carried out before the child reaches 10 years of age [9]. Scoliosis usually develops at a later age [13]. Those children who developed WS after 10 years of age had either isolated WS or WS in combination with scoliosis.

Letts et al. [1] analyzed the temporal relationship of hip dislocation, scoliosis, and pelvic obliquity in the development of WS. In their series, three out of four cases started with hip dislocation, followed by pelvic obliquity and, finally, scoliosis. In the present study, only one child participating in the hip surveillance program developed hip dislocation, and there was a significantly lower frequency of WS starting in the lower extremities in the study group. A knee contracture might cause tilting of the legs to one side in a supine position, and start the development of WS. In addition to preventing hip dislocation, early identification and treatment of hip and knee contractures could be an explanation for the reduced incidence of WS in the study group. A previous study including the same age cohorts as in the present study showed that the range of passive movement in hips, knees, and ankles improved significantly in non-ambulant children born in 1992–1995 compared with those born in 1990–1991 [2].

Three hips were defined as exhibiting WS after unilateral femoral varus osteotomy to prevent hip dislocation. Some authors recommend bilateral osteotomy in children with unilateral displacement [14, 15]. However, Owers et al. [16] presented a series of 30 children with WS who were treated with bilateral femoral osteotomy and soft tissue release to gain symmetry. At the 3-year follow-up, 44 % of the children had a WS deformity compared with 50 % preoperatively. Thus, it is uncertain whether bilateral femoral osteotomy reduces the long-term risk of developing WS.

Early treatment of scoliosis might reduce the development of pelvic obliquity and WS. With improved knowledge of the risk factors for progression and new surgical techniques that allow for further growth, this might be one way to reduce WS in the future.

In conclusion, WS is a severe problem, affecting about one-third of children with CP at GMFCS levels III–V. In most children, WS develops before 10 years of age, but the risk continues up to 20 years of age. With early inclusion in a hip surveillance program, and early treatment of contractures, the frequency of WS starting in the lower extremities can be reduced.
